# A Novel Loop-Mediated Isothermal Amplification (LAMP) Primer Set for Detecting the STY2879 Gene of *Salmonella enterica* Serovar Typhi in Raw Milk

**DOI:** 10.3390/microorganisms14020297

**Published:** 2026-01-27

**Authors:** Hyuck-Jin Seo, Timothy E. Riedel

**Affiliations:** Freshman Research Initiative, DIY Diagnostics Laboratory, College of Natural Sciences, The University of Texas at Austin, Austin, TX 78712, USA; justin.seo244@utexas.edu

**Keywords:** *Salmonella*, *enterica*, serovar, Typhi, typhoid fever, unpasteurized, raw milk, loop-mediated isothermal amplification, LAMP, diagnostic, field test, detection, STY2879, primer set

## Abstract

Milk-borne outbreaks remain a significant issue in low-income countries due to unhygienic practices. Currently, there are no known easily accessible and low-cost diagnostic tests that can detect *Salmonella enterica* subsp. *enterica* serovar Typhi, the causative agent of typhoid fever, in raw milk samples at high specificity, sensitivity, and speed without preprocessing. Early detection of *Salmonella enterica* subsp. *enterica* serovar Typhi in food matrices is critical for preventing infection prior to consumption and reducing disease burden. Using colorimetric loop-mediated isothermal amplification, we screened 15 novel and two previously published primer sets. We identified one novel primer set capable of detecting the STY2879 gene in as few as 2000 genomes in 2% v/v raw milk reactions and 1000 genomes in 1% v/v raw milk reactions of *Salmonella enterica* subsp. enterica serovar Typhi after 30 minutes of incubation in a 65° C water bath. The colorimetric readout offers promising potential applications for on-site detection in remote and low-resource settings where infection with *Salmonella enterica* subsp. *enterica* serovar Typhi remains a public health concern.

## 1. Introduction

*Salmonella enterica* subsp. *enterica* serovar Typhi (*S*. Typhi) is a highly pathogenic, gram-negative, human-restricted bacterium responsible for typhoid fever, a systemic illness characterized by fever, headache, diarrhea, abdominal pain, and, in severe cases, death [1]. Globally, in 2017, *S*. Typhi infected approximately 10.9 million people and resulted in nearly 120,000 deaths [2]. Transmission typically occurs through the ingestion of contaminated food or water, and unpasteurized raw milk has increasingly been recognized as a vehicle of transmission. A recent study from Ecuador analyzed 600 raw milk samples and reported a 37.5% prevalence of *Salmonella enterica* contamination. Among the isolates, 28 were identified as *S*. Typhi, and 19.1% of the *Salmonella*-positive samples could not be assigned a specific serovar, suggesting that the true prevalence of *S*. Typhi contamination may be even higher than reported in raw milk [3].

In developed and high-income countries (HICs), the burden of global milk-borne outbreaks is low, as nearly all milk is pasteurized and maintained under food safety standards [4]. However, in low-income countries (LICs) such as Kenya, Ethiopia, and other East African countries, the burden of milk-borne outbreaks remains high. Farmers were found to lack adequate training in milk safety standards, and milking conditions were unhygienic as the majority did not engage in teat dipping practices and used plastic containers and untreated water [5]. Furthermore, pastoral communities in such LICs were strongly opposed to participating in pasteurization practices due to a misconception that pasteurization would deplete the milk of its nutrients. As a result, pastoralists were largely found to consume unpasteurized raw milk from markets and directly from animal udders [6].

Currently, there are no known readily available diagnostic tests that can accurately detect *S*. Typhi in raw milk samples with the high specificity, sensitivity, speed, and low cost needed to be effective in LICs. Loop-mediated isothermal amplification (LAMP) is a broadly adopted nucleic acid amplification method that has the potential to overcome such shortcomings [7,8]. While similar to PCR, LAMP reactions do not require thermocyclers and can be carried out using a simple constant-temperature water bath or heater device [9]. LAMP reactions utilize four to six primers, allowing for rapid amplification while maintaining a high level of specificity. This makes LAMP a strong candidate for diagnostic applications in developing and LICs with limited resources [10]. In addition, ready-to-use colorimetric LAMP systems are available that allow for easy end-user color-change interpretation. Colorimetric LAMP assays sometimes have issues with nonspecific amplification products developing during the incubation period, which can lead to false positives [11]. This problem can be mitigated with additional error-checking tools, such as those presented by Jiang et al. [12], but these often lead to increased complexity of the diagnostic systems and reduce the low-resource advantage.

In this study, we aimed to develop a field-deployable colorimetric LAMP assay for the detection of *S*. Typhi in raw milk as a preventive food safety intervention to reduce *S*. Typhi infection, mitigate the burden of milk-borne outbreaks, and promote public health in LICs. To accomplish this, we systematically evaluated existing and newly designed primer sets for delayed nonspecific amplification, a critical determinant of assay reliability in colorimetric LAMP systems. We screened two previously published primer sets and 15 novel primer sets using the NEB colorimetric LAMP system and quantified their performance based on time to full amplification in the absence of positive controls (PCs). Primer sets exhibiting the longest delays in full nonspecific amplification were further evaluated by measuring the time differential between full negative and positive control amplification (defined in this study as differential time to threshold or DTT). A larger DTT indicates a lower risk of false-positive interpretation. The primer set with the highest DTT was subsequently assessed for robustness at lower signal concentrations and with the direct addition of whole and raw milk.

## 2. Materials and Methods

### 2.1. Primer Sets

**Figure 1:**
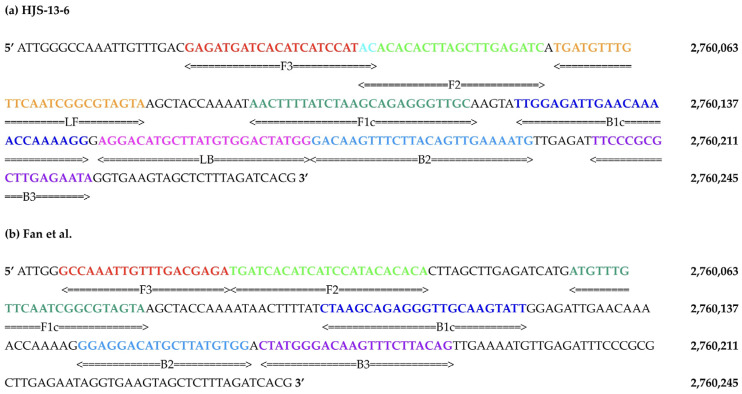
Comparison of primer binding sites between the (**a**) newly designed HJS-13-6 primer set and the (**b**) Fan et al. [14] primer set. The template sequence used for designing both sets is a segment within the STY2879 gene (shown 5′ to 3′). Colored regions represent individual primer binding sites: F3 (in red), FIP (in dark and light green), BIP (in dark and light blue), and B3 (in purple). The HJS-13-6 primer set includes a two-base-pair overlap (in cyan) between the F3 and F2 regions that enables the NEB LAMP Primer Design Tool [17] to generate loop primers: LF (in orange) and LB (in pink). Nucleotide numbering corresponds to the *S*. Typhi strain CT-18 genome (NCBI Reference Sequence: NC_003198.1).

Two previously reported LAMP primer sets, denoted Abdullah et al. [13] and Fan et al. [14], and 15 novel primer sets (Table 1, Table S1), denoted by the prefix HJS, were evaluated in this study. The novel primer sets were designed to target a validated [14,15,16] segment within the STY2879 target gene (Figure 1) using the NEB LAMP Primer Design Tool Version 1.4.2 [17]. Two base pairs were added to the segment to improve the likelihood of loop primer design (Table S2). All LAMP primers were designed following NEB-recommended parameters (Table 2) optimized for assay robustness, specificity, and reaction speed in the NEB colorimetric LAMP system at a reaction incubation temperature of 65 °C [18].

### 2.2. Colorimetric LAMP Assay Construction

We utilized a colorimetric LAMP system as a qualitative measure for the amplification of the target DNA sequence (New England BioLabs, Ipswich, MA, USA, cat. # M1800L). All water used in this study was RNase-, DNase-, and protease-free molecular biology grade water (Corning, Manassas, VA, USA; cat. #46-000-CM). All LAMP reactions described in this study, including those that utilize primers from Abdullah et al. [13] and Fan et al. [14], followed the recommended settings (reaction temperature and primer concentrations) outlined in the WarmStart Colorimetric LAMP 2X Master Mix Typical LAMP Protocol (details described below). In 0.2 mL tubes, 25 μL reactions were constructed with the following: 12.5 µL of NEB 2X Colorimetric Master Mix (contains all necessary ingredients for colorimetric LAMP except primers and control inocula), 8 μL water, and 2.5 μL of a 10X mix of all primers (stored frozen until use) for final primer concentrations of 0.2 μM forward outer primer (F3) and backward outer primer (B3), 1.6 μM forward inner primer (FIP, composed of F1c and F2 regions) and backward inner primer (BIP, composed of B1c and B2 regions), and 0.4 μM loop forward (LF) and loop backward (LB). The above components (Table 3) bring the volume in each reaction tube to 23 μL. The final two microliters were added prior to incubation and were used for negative and positive control inoculations, as described in Section 2.3.

### 2.3. Controls

For non-template negative controls (NCs), the final two microliters were used to inoculate reactions with a sample matrix of either water, 25% v/v diluted cow’s whole milk, or 25% v/v diluted cow’s raw milk. Dilutions were performed on the milk matrices using water on the same day the experimental trials were conducted.

In some positive controls (PCs), the final two microliters of the reaction were used to inoculate reactions with synthetic double-stranded DNA (gBlock, Integrated DNA Technologies) of the STY2879 target gene fragment (Table S2). The dehydrated double-stranded synthetic DNA was originally resuspended with Tris-EDTA buffer pH 8.0 (Fisher Scientific, Waltham, MA, USA) to a concentration of 10^10^ copies of target gene fragments/μL and stored frozen at −20 °C in multiple aliquots. Before each experimental trial that utilized target gene fragments, serial dilutions were performed with the corresponding NC sample matrix on a previously unused thawed aliquot of 10^10^ copies of target gene fragments/μL to achieve a final inoculum concentration of either 10^5^ or 10^3^ copies of target gene fragments/μL.

In later PCs to further validate the HJS-13-6 primer set, the final two microliters of the reaction were used to inoculate reactions with complete genomic DNA isolated from *S*. Typhi strain Ty2 (GenBank: AE014613.1; BEI Resources, NIAID, NIH: Genomic DNA from *Salmonella enterica* subsp. *enterica* serovar Typhi strain Ty2, NR-543). The genomic DNA was resuspended with TE buffer to a concentration of 10^7^ genome copies/μL and stored frozen at −20 °C in multiple aliquots. Before each experimental trial that utilized genomic DNA, serial dilutions were performed with the corresponding NC sample matrix on a previously unused thawed aliquot of 10^7^ genome copies/μL to achieve a final inoculum concentration of 10^3^ genome copies/μL. Some reactions were spiked with 2 μL of this inoculum to achieve a final reaction containing 2000 genomes in 2% v/v milk. Other reactions were spiked with 1 μL of the inoculum and 1 μL of water to achieve a final reaction containing 1000 genomes in 1% v/v milk.

### 2.4. Incubating LAMP Reactions and Scoring Results

**Figure 2:**
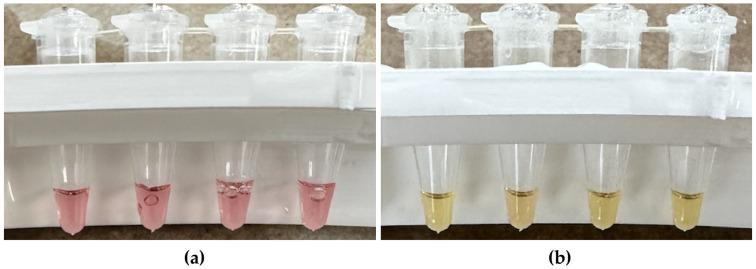
Photographs illustrating the colorimetric response of four parallel LAMP reactions during a representative screening experimental trial. The tubes shown are non-template negative control (NC) replicate reactions from a single experimental trial evaluating the performance of primer set HJS-13-4 in a water matrix containing 10^5^ copies of DNA/μL of the STY2879 gene fragment (Figure 3). Images were captured after temporarily removing the tubes from incubation in a 65 °C water bath at defined time points. The left panel (**a**) was taken after 10 minutes of incubation, when all reactions remained pink, indicating no detectable amplification. The right panel (**b**) was taken after 70 minutes of incubation, when all reactions had turned yellow, indicating full DNA amplification.

Strips of qPCR tubes containing the reaction mixtures were inserted in a water bath float and incubated in a laboratory water bath set to 65 °C. Reactions were analyzed every 10 minutes of incubation by removing the float from the water bath, photographing the reactions (Figure 2), and then returning the float to the water bath, taking approximately 10 seconds. Reaction colors were scored with the unaided eye as either pink, indicating no amplification; orange, indicating intermediate levels of amplification; or yellow, indicating full amplification until all the reactions reached a yellow terminal endpoint. These LAMP reactions change color from pink to yellow due to pH changes resulting from DNA amplification.

## 3. Results

### 3.1. Initial Screenings of Primer Sets

**Figure 3:**
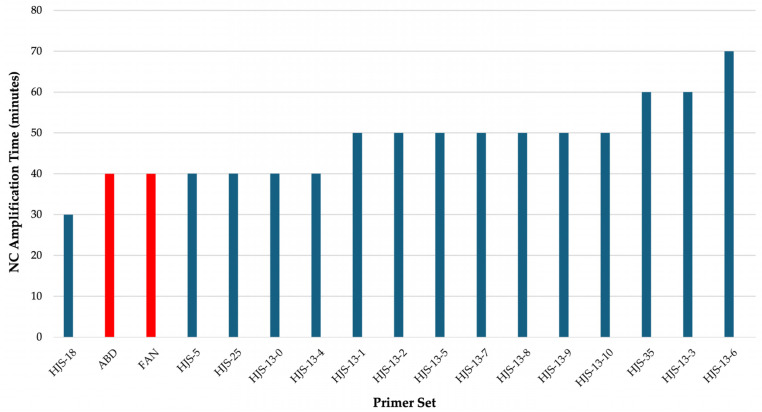
Non-template negative control (NC) amplification times. Each bar in the chart represents the median of at least 4 replicates of a primer set tested in a water matrix without positive controls (PCs). The blue bars represent newly designed primer sets (this study, Table 1 and Table S1) and the red bars represent previously published primer sets, Abdullah et al. [13] and Fan et al. [14].

To evaluate the performance of candidate primer sets targeting the STY2879 gene, an initial comparative screening was conducted using only NC reactions prepared in a water matrix (Figure 3). This screening was designed to assess nonspecific amplification in the absence of target DNA. Primer sets that remained negative (pink) for the longest duration were considered to have the slowest nonspecific amplification. The primer sets with longest median full NC amplification time were HJS-13-6, HJS-13-3, and HJS-35. Primer set HJS-13-6 exhibited a full NC amplification time of 70 minutes, and primer sets HJS-13-3 and HJS-35 exhibited a full NC amplification time of 60 minutes. These three primer sets were selected for further screening.

**Figure 4:**
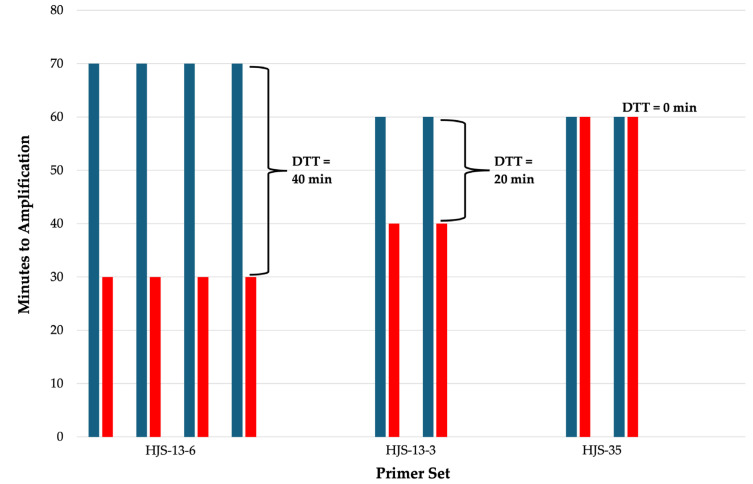
Differential time to thresholds (DTT) of the three primer sets with the longest full NC amplification times. DTT is the difference in full amplification times of NC (blue bars) and PC reactions (red bars). Longer DTTs are desirable for LAMP assays. Reactions for a given primer set were tested in a water matrix containing 10^5^ copies/μL of the STY2879 gene fragment. Braces demonstrate the DTT value between corresponding NC and PC reactions.

Primer sets HJS-13-6, HJS-13-3, and HJS-35 were subsequently evaluated for amplification efficiency and discrimination capability using the differential time to threshold (DTT) as the primary performance metric. DTT is defined in this study as the time difference between the onset of full NC amplification and PC amplification. For each primer set, reactions containing a defined concentration of the STY2879 gene fragment as the PC and corresponding NC reactions were run in parallel under identical reaction conditions. The onset time of visible color change in each reaction was recorded, and DTT values were calculated accordingly. The three primer sets were compared in a water matrix using the STY2879 gene fragment as the PC at a concentration of 10^5^ copies of DNA/μL. Primer set HJS-13-6 exhibited the greatest DTT of 40 minutes, followed by HJS-13-3 with a DTT of 20 minutes, and primer set HJS-35 showed no ability to discriminate PCs from NCs with a DTT of 0 minutes (Figure 4).

### 3.2. Validation of Novel Primer Set HJS-13-6

In the initial screenings of primer sets, HJS-13-6 exhibited the greatest time to full nonspecific amplification and the greatest DTT of 40 minutes when spiking the PCs with the STY2879 gene fragment at a concentration of 10^5^ copies of DNA/μL in a water matrix. Based on this performance, HJS-13-6 was selected for further validation using different positive controls, PC concentrations, and sample matricies (Table 4).

When the STY2879 gene fragment was used as the PC, spiking PCs with a lower concentration of 10^3^ DNA copies/μL of the STY2879 fragment in a water matrix resulted in a DTT of 30 minutes. No amplification was observed in PCs containing 8% or 4% v/v whole milk reaction matrices. However, amplification was detected in a 2% v/v whole milk reaction matrix with a DTT of 20 minutes. Replacing the 2% v/v whole milk reaction matrix with a 2% v/v raw milk reaction matrix increased the DTT to 30 minutes.

Primer set HJS-13-6 was then further tested with the more authentic positive control of complete genomic DNA isolated from *S*. Typhi strain Ty2. At a concentration of 2000 genome copies/reaction in a 2% v/v raw milk reaction matrix, a DTT of 30 minutes was observed. At a concentration of 1000 genome copies/reaction in a 1% v/v raw milk reaction matrix, a DTT of 20 minutes was observed.

## 4. Discussion and Conclusions

Due to the NEB colorimetric LAMP system’s potential for use in low-resource settings, we screened 17 primer sets targeting *S*. Typhi for their performance in this system. Fifteen novel primer sets were designed to target a segment of the STY2879 gene of *S*. Typhi originally identified by Fan et al. [14] to be specific to *S*. Typhi and highly conserved across *S*. Typhi isolates.

Fan et al. [14] established the STY2879 gene through multi-step comparative genomics and experimental validation using DNA from 22 geographically and temporally diverse *S*. Typhi isolates and 75 non-typhoidal *Salmonella* strains. From the process, the STY2879 gene emerged as a locus showing 100% positivity in all tested *S*. Typhi isolates and zero cross-reactivity with non-Typhi strains.

Subsequent independent clinical studies have further validated the diagnostic reliability of this target. Kaur et al. [15] incorporated STY2879-targeted primers into their magnetic nanoparticle-based “Miod” diagnostic platform for *S*. Typhi detection and observed no cross-reactivity with common bloodstream pathogens such as *Escherichia coli*, *Staphylococcus aureus*, *Pseudomonas aeruginosa*, *Acinetobacter baumannii*, *Enterococcus faecalis*, *Salmonella* Paratyphi A, and *Klebsiella pneumonia* at concentrations as high as 10^6^ CFU/mL in 28 clinical blood samples. In a more recent clinical study, Heamchandsaravanan et al. [16] confirmed the diagnostic performance of the STY2879 gene across 107 clinical blood samples.

To further support these prior findings, we conducted an in silico comparative analysis to assess the conservation of the STY2879 target gene segment used in this study. The inner primer regions (F2 and B2) are the first sequences to hybridize and initiate strand displacement synthesis. They define the core LAMP target and are considered the key determinant of reaction specificity and efficiency [7]. Accordingly, the F2-B2 region was selected for alignment and comparative analysis. A BLASTN [19] analysis using the F2-B2 sequence was conducted against complete genome assemblies of *S*. Typhi (taxid: 90370). All 172 available complete *S*. Typhi genome assemblies exhibited 100% sequence identity to the F2-B2 region, indicating conservation of the LAMP core across all sequenced *S*. Typhi strains (Table S3).

Collectively, the combination of previous experimental validation, independent clinical studies [14,15,16], and our in silico analysis demonstrates that STY2879 is a highly conserved and *S*. Typhi-specific target. The conservation of the F2-B2 inner primer region across all available *S*. Typhi genome assemblies supports its suitability for LAMP-based detection, reinforcing the robustness of the STY2879 gene segment used in this study as an effective molecular target for rapid and accurate detection of *S*. Typhi with the HJS-13-6 assay.

Since LAMP primer sets can be hampered by nonspecific amplification, the 15 novel primer sets and the primers from Fan et al. [14] and Abdullah et al. [13] were initially screened for the duration before full nonspecific amplification occurred in the NEB system when no PCs were added (Figure 3). Three of the new primer sets showed a longer delay to full nonspecific amplification of at least 60 minutes when compared to either Fan et al. [14] or Abdullah et al. [13]. In the NEB system, both Fan et al. [14] and Abdullah et al. [13] primer sets showed that all NC reactions amplified fully within 40 minutes. This discrepancy may be due to differences in primer design, incompatibility of the primers with the NEB colorimetric LAMP reaction conditions, or a lack of optimization of reaction components [18].

The three primer sets with the longest delays to full NC amplification were further screened with positive controls We found set HJS-13-6 had the largest DTT, time window between full negative and positve control amplification. Using DTT as a comparative metric provides a quantitative and standardized measure of how rapidly each primer set amplified true target DNA relative to any background signal, enabling objective selection of the most effective primer set for detecting the STY2879 target gene. Primer sets exhibiting larger DTT values were interpreted as indicating faster and more robust amplification relative to background signal and as both efficient (rapid PC amplification) and specific (delayed or absent NC amplification).

Primer set HJS-13-6 was the most effective in detecting the STY2879 target gene in a water matrix using the STY2879 gene as the PC at a concentration of 10^5^ copies of DNA/μL. HJS-13-6 features overlapping F3 and F2 primer sites, which enabled the target segment to be suitable for incorporating loop primers into the design. The two-base-pair overlap occurs at the 5′ end of the F2 region that does not participate in primer extension. Because FIP initiation relies on the 3′ end of F2 and tolerates a long 5′ overhang, this overlap is not expected to significantly affect LAMP kinetics or specificity. Any potential negative impact appears to be offset by the performance enhancement provided by the addition of the loop primers [20]. This is consistent with the displacement properties of Bst polymerases used in LAMP [21].

HJS-13-6 was further characterized for the limit of detection and tolerance for the direct addition of milk to the reaction. HJS-13-6 demonstrates the ability to detect low concentrations of STY2879 even when the final reaction volume is 2% and 1% v/v milk (Table 5). To the best of our knowledge, this is the first study to demonstrate a successful *S*. Typhi LAMP assay carried out with raw milk in the reaction matrix at a concentration of 2% and 1% v/v. The sensitivity of HJS-13-6 in the NEB system is estimated to be 2000 genomes in 2% v/v raw milk reactions and 1000 genomes in 1% v/v raw milk reactions of *Salmonella enterica* subsp. *enterica* serovar Typhi. This crude limit of detection estimation is based on the user having at least 30 minutes to interpret the color output.

Fan et al. [14] and Abdullah et al. [13] report sensitivities of 15 copies/reaction and 20 CFU/reaction, respectively. HJS-13-6 in its current form is less sensitive than Fan et al. [14] and Abdullah et al. [13], but this limitation may be mitigated in three ways. First, the HJS-13-6 assay is not fully optimized with regard to reactant concentrations and reaction temperatures. Optimization can often lead to a 10–100-fold greater sensitivity [22]. Second, high assay sensitivity is not always critical for diagnostic test effectiveness, depending on the health thresholds and sample preparation steps. For example, Riyaz-Ul-Hassan et al. [23] found at least one raw dairy milk sample from India with a *Salmonella* concentration of ~10^5^ cells/1 mL of milk. Additionally, samples are often concentrated or enriched before being moved into the LAMP reaction, thus increasing the initial *Salmonella* levels by orders of magnitude [14]. Finally, the sensitivity of HJS-13-6 is resistant to the input of milk (Table 5), indicating robustness to any carryover of milk during sample preprocessing. This robustness may enable simplified sample processing and minimal wash steps after filtering, ultimately increasing the usability of the system in a low-resource setting in spite of the lower sensitivity.

The ability of HJS-13-6 to quickly detect STY2879 in various reaction conditions (Table 5) warrants further studies of this primer system. This study, Fan et al. [14], and additional studies [15,16] confirmed that STY2879 is a highly specific and conserved diagnostic target for *S*. Typhi. Future work should focus on improving the sensitivity of HJS-13-6 in the NEB system and conducting additional *in vitro* specificity tests of diverse *Salmonella* serovars and strains and other common bacteria and pathogens that occur in raw milk, such as *Streptococcus thermophilus*, *Lactococcus lactis*, *Campylobacter* spp., *Escherichia coli*., *Listeria monocytogenes*, *Yersinia* spp., *Coxiella burnetii*, *Brucella* spp., and *Mycobacterium* spp. This study only included the addition of DNA directly to the assays and therefore does not address any complications that may come from a bacterial cell lysis or preamplification step. This critical part of the sample-to-answer process needs to be addressed in future research for proactive infection prevention.

The novel HJS-13-6 primer set identified in this study is a first step in the development of a rapid *S*. Typhi detection test for raw milk samples prior to consumption to strengthen preventive measures against infection. Due to the low resource demand of the colorimetric NEB LAMP system, primer set HJS-13-6 has promising potential to reduce the burden of milk-borne outbreaks and preserve public health in raw milk-consuming communities in LICs.

## Figures and Tables

**Table 1:** Primer sequences of newly designed loop-mediated isothermal amplification (LAMP) primer set HJS-13-6 (this study) and previously published primer sets from Abdullah et al. [13] and Fan et al. [14], reported in the 5′ to 3′ direction. HJS-13-6 and Fan et al. [14] primers were designed to target the STY2879 gene, while Abdullah et al. [13] primers were designed to target the STBHUCCB_38510 gene. Sequences of the remaining 14 newly designed HJS primer sets are provided in Table S1.

	HJS-13-6	Abdullah et al. [13]	Fan et al. [14]
F3	GAGATGATCACATCATCCATAC	TCTGGCACTCCTGTGCCTT	GCCAAATTGTTTGACGAGA
B3	TATTCTCAAGCGCGGGAA	GCTCAAGACGAGAAACAGG	CTGTAAGAAACTTGTCCCATAG
FIP (F1c-F2)	GCAACCCTCTGCTTAGATAAAAGTT-ACACACACTTAGCTTGAGATC	TAGAAATAGTAGAGTCAGG-GCTTTTGCAGGTATTGTGG	TACTACGCCGATTGAACAAACAT-TGATCACATCATCCATAAACACA
BIP (B1c-B2)	TTGGAGATTGAACAAAACCAAAAGG-CATTTTCAACTGTAAGAAACTTGTC	TTGCCTTATCTAATACAAG-GTAAAAGGTGGTTTGCTCT	CTAAGCAGAGGGTTGCAAGTATT-CCACATAAGCATGTCCTCC
LF	TACTACGCCGATTGAACAAACATCA	ATGAAAAATGACGCGAGTT	None
LB	AGGACATGCTTATGTGGACTATGG	GTTGATCCTTTCAGTAAGG	None

**Table 2:** LAMP primer design parameters used to generate 15 novel primer sets evaluated in this study. These parameters enforce optimal amplicon length, loop primer melting temperature, controlled GC content, terminal stability, and minimized secondary structure formation to maximize assay speed, sensitivity, and specificity in the NEB colorimetric LAMP system at a reaction incubation temperature of 65 °C.

Category	Parameter	Value/Range
Reaction Conditions (mM)	Na^+^ concentration	50
	Mg^2+^ concentration	8
Primer Length (bp)	F1c/B1c	20–22
	F2/B2	18–20
	F3/B3	18–20
	LF/LB	15–25
Melting Temperature (°C)	F1c/B1c	64–66
	F2/B2	59–61
	F3/B3	59–61
	LF/LB	64–66
GC Content (%)	All primers	40–65
	Loop primers	40–65
Terminal Stability (ΔG, kcal·mol^−1^)	5′ end	≤−4
	3′ end	≤−4
	3′ end (loop primers)	≤−2
Secondary Structure Thresholds (ΔG, kcal·mol^−1^)	Dimer check	≤−2.5
	Dimer check (loop primers)	≤−3.5
Inter-Primer Distances (bp)	F2-B2 (amplicon length)	120–160
	F1c-F2 (loop region)	40–60
	F2-F3	0–60
	F1c-B1c	0–100
Primer Count Limits	F1c/B1c	≤3
	F2/B2	≤10
	F3/B3	≤3
	LF/LB	≤10

**Table 3:** Stock and final concentrations of primers for all LAMP reactions reported in this study.

Primer	10X Stock (μM)	1X Reaction (μM)
F3	2	0.2
B3	2	0.2
FIP	16	1.6
BIP	16	1.6
LF	4	0.4
LB	4	0.4

**Table 4:** Amplification of primer set HJS-13-6 using different PC types (Ty2 indicates copies pf complete genomes isolated from *S*. Typhi strain Ty2; STY indicates copies of synthetic STY2879 gene fragments), PC concentrations, and reaction matrices. Reaction colors were scored with the unaided eye as either pink, indicating no amplification; orange, indicating intermediate levels of amplification; or yellow, indicating full amplification until all the reactions have turned yellow (Figure 2). Color outcomes shown in the table represent the median result across replicate reactions (RXNs) for each reaction condition tested. All replicate reactions within each condition produced consistent amplification with no variation.

	2000 Ty2/RXN in 2% Raw Milk		1000 Ty2/RXN in 1% Raw Milk		2000 STY/RXN in 2% Raw Milk		2000 STY/RXN in 2% Whole Milk		2000 STY/RXN in H_2_O		200,000 STY/RXN in H_2_O	
Control	PC	NC	PC	NC	PC	NC	PC	NC	PC	NC	PC	NC
**Replicate Number**	10	10	3	3	2	2	6	6	5	5	4	4
**10 min**												
**20 min**												
**30 min**												
**40 min**												
**50 min**												
**60 min**												
**70 min**												

**Table 5:** Effectiveness of the novel primer set HJS-13-6 across different reaction conditions measured by DTT. Larger DTT values indicate a better-performing assay.

PC	PC Concentration	Matrix	DTT (min)
Genomic DNA Isolated from *S*. Typhi strain Ty2	2000 genomes/reaction	2% raw milk	30 ± 5
Genomic DNA Isolated from *S*. Typhi strain Ty2	1000 genomes/reaction	1% raw milk	20 ± 5
Synthetic STY2879	2000 fragments/reaction	2% raw milk	30 ± 5
Synthetic STY2879	2000 fragments/reaction	2% whole milk	20 ± 5
Synthetic STY2879	2000 fragments/reaction	H_2_O	30 ± 5
Synthetic STY2879	200,000 fragments/reaction	H_2_O	40 ± 5

## Data Availability

The original contributions presented in this study are included in the article/Supplementary Material. Further inquiries can be directed to the corresponding author.

## Supplementary Materials

The following supporting information can be downloaded at: https://www.mdpi.com/article/10.3390/microorganisms14020297/s1, Table S1. Primer sequences of 15 novel LAMP primer sets designed for the STY2879 target gene segment, reported in the 5’ to 3’ direction.; Table S2. Sequence of the STY2879 target gene segment used in this study.; Table S3: BLASTN analysis of the F2-B2 region of the STY2879 target gene segment across complete *S*. Typhi genome assemblies, showing percent sequence identity for each strain.

## Abbreviations

The following abbreviations are used in this manuscript:

DTT	Differential time to threshold (time difference full between negative control and positive control amplification)
LAMP	Loop-mediated isothermal amplification
F3	Forward outer primer
B3	Backward outer primer
FIP	Forward inner primer (composed of F1c and F2 regions)
BIP	Backward inner primer (composed of B1c and B2 regions)
LF	Loop forward primer
LB	Loop backward primer
PC	Positive control
NC	Non-template negative control
RXN	Reaction
